# Expression Profiling of FSHD-1 and FSHD-2 Cells during Myogenic Differentiation Evidences Common and Distinctive Gene Dysregulation Patterns

**DOI:** 10.1371/journal.pone.0020966

**Published:** 2011-06-13

**Authors:** Stefania Cheli, Stephanie François, Beatrice Bodega, Francesco Ferrari, Elena Tenedini, Enrica Roncaglia, Sergio Ferrari, Enrico Ginelli, Raffaella Meneveri

**Affiliations:** 1 Department of Biology and Genetics for Medical Sciences, University of Milan, Milan, Italy; 2 Department of Experimental Medicine, University of Milano-Bicocca, Monza, Italy; 3 Department of Biomedical Sciences, University of Modena and Reggio Emilia, Modena, Italy; Université Paris-Diderot, France

## Abstract

**Background:**

Determine global gene dysregulation affecting 4q-linked (FSHD-1) and non 4q-linked (FSHD-2) cells during early stages of myogenic differentiation. This approach has been never applied to FSHD pathogenesis.

**Methodology/Principal Findings:**

By *in vitro* differentiation of FSHD-1 and FSHD-2 myoblasts and gene chip analysis we derived that gene expression profile is altered only in FSHD-1 myoblasts and FSHD-2 myotubes. The changes seen in FSHD-1 regarded a general defect in cell cycle progression, probably due to the upregulation of myogenic markers PAX3 and MYOD1, and a deficit of factors (SUV39H1 and HMGB2) involved in D4Z4 chromatin conformation. On the other hand, FSHD-2 mytubes were characterized by a general defect in RNA metabolism, protein synthesis and degradation and, to a lesser extent, in cell cycle. Common dysregulations regarded genes involved in response to oxidative stress and in sterol biosynthetic process. Interestingly, our results also suggest that miRNAs might be implied in both FSHD-1 and FSHD-2 gene dysregulation. Finally, in both cell differentiation systems, we did not observe a gradient of altered gene expression throughout the 4q35 chromosome.

**Conclusions/Significance:**

FSHD-1 and FSHD-2 cells showed, in different steps of myogenic differentiation, a global deregulation of gene expression rather than an alteration of expression of 4q35 specific genes. In general, FSHD-1 and FSHD-2 global gene deregulation interested common and distinctive biological processes. In this regard, defects of cell cycle progression (FSHD-1 and to a lesser extent FSHD-2), protein synthesis and degradation (FSHD-2), response to oxidative stress (FSHD-1 and FSHD-2), and cholesterol homeostasis (FSHD-1 and FSHD-2) may in general impair a correct myogenesis. Taken together our results recapitulate previously reported defects of FSHD-1, and add new insights into the gene deregulation characterizing both FSHD-1 and FSHD-2, in which miRNAs may play a role.

## Introduction

Facioscapulohumeral muscular dystrophy (FSHD [OMIM 158900]) is the third most frequent form of muscle diseases, inherited as an autosomal dominant trait, with an estimated incidence of 1 in 20,000.

The disease is predominantly characterized by progressive, often asymmetric, weakness and wasting of facial, shoulder and upper arm muscles [1]. Interfamilial and intrafamilial variability, with severity ranging from asymptomatic carriers (20% of individuals related to FSHD patients) to loss of ambulation, are also described [2]–[3]. Males are on average more often and more severely affected than females [4].

The molecular defect associated to the disorder has been mapped to the subtelomeric region of the long arm of chromosome 4 (4q35) where a large, complex macrosatellite (the D4Z4 repeat array) is present [5]. In the general population, the D4Z4 repeat array is polymorphic and it may vary from 11 to more than 100 units of 3.3 kb, whereas most of FSHD patients (FSHD-1) carry only 1 to 10 repeat units [6]. To develop FSHD, D4Z4 contraction needs to occur on a specific genetic background; in fact, only contractions associated with some chromosome 4 variants, such as the 4qA161 and the newly discovered uncommon 4qA159 and 4qA168, are permissive [7]–[8]. It is noticeable that monosomy of 4qter or entire deletions of D4Z4 repeat array are not associated with the disorder, so a critical role for this genomic region and its flanking sequences in FSHD pathogenesis is to be expected.

However, a small percentage of FSHD cases (<5%) (defined FSHD-2 patients), shows at least one 4qA161 chromosome but no contraction of 4q35 D4Z4 [9]–[10]. This subset of patients appears very heterogeneous and to date no disease locus has been identified.

Furthermore, recent studies showed that FSHD-1 and FSHD-2 patients are characterized by 4q D4Z4 hypomethylation that is contraction-dependent in FSHD-1 and contraction-independent in FSHD-2 patients [10]–[11]. Current models of FSHD pathogenesis suggest that D4Z4 contraction (FSHD-1) or other not yet known genetic defects (FSHD-2), results in chromatin modification that could generate aberrant expression of a putative gene encoded by the D4Z4 repeat, termed double-homeobox 4 (*DUX4*) [8], [12]–[14], or of genes in cis to the D4Z4 array [15], or elsewhere in the genome (in trans). However, until now disagreement remains on whether single genes are reliably mis-expressed or causative for FSHD.

One approach with no an *a priori* model on the molecular basis of the disease is represented by the derivation of global gene expression profile in cells derived from affected patients in comparison to control ones. Although several transcriptome studies have been published on FSHD-1, only one was carried out on primary myoblasts [16], and none has considered gene variations in different steps of myogenic differentiation. Furthermore, no studies have been previously reported on global gene expression in FSHD-2.

In this paper, we present global gene-expression profiles of myoblasts from FSHD-1 and FSHD-2 patients and healthy controls in the context of myogenic differentiation.

## Materials and Methods

### Cell lines and patients

Human primary myoblasts derived from FSHD-1 and FSHD-2 (non 4q-linked or phenotypic FSHD) patients and from healthy controls were obtained from the Telethon BioBank (Neuromuscular Disease and Neuroimmunology Unit, Muscle Cell Biology Laboratory, C. Besta Neurological Institute). Table 1 reports the main features of the used cell lines. Cells were grown in Dulbecco's Modified Eagle Medium (DMEM) containing 20% fetal bovine serum (FBS), L-glutamine (1%), penicillin and streptomycin (1%) (Euroclone), insulin 10 mg/ml (Sigma), human fibroblast growth factor (hFGF) 25 ng/ml and human epidermal growth factor (hEGF) 10 ng/ml (Peprotech). Myotubes were obtained after treatment in DMEM supplemented with 2% horse serum (Euroclone) and 1% insulin (Sigma), for 8 days (differentiating medium) [17]. All experiments were performed using cell lines between 2 and 10 population doubling (PD) to avoid premature replicative senescence which normally occurs after 10–15 PD (Table 1). All FSHD patients satisfied the accepted clinical criteria for FSHD. FSHD-1 had undergone DNA diagnosis and were identified as carriers of small (<38 kb, <11 repeats) 4q35-located D4Z4 repeat arrays, as determined by p13E-11 hybridization to *EcoRI*-digested and *EcoRI/BlnI*-digested genomic DNA (Table 1). FSHD-2 patients were considered those showing: a) FSHD clinical signs [18] and b) a 4q D4Z4 cluster size higher than 38 kb (Table 1). Regarding the clinical signs, FSHD-2 S1 presented with severe shoulder (involving trapezius, arm rotator and extension muscles) and pelvic (mainly gluteus) girdle weakness, as well as a marked facial weakness. FSHD-2 S2 presented with severe shoulder girdle weakness (inability to lift arms above shoulder level), orbicularis oculi and orbicularis oris weakness and modest pelvic girdle weakness. Furthermore, both FSHD-2 patients were subjected to molecular analysis on Calp-3 and dysferlin gene products (molecular markers of the two main forms of Limb girdle dystrophies: LGMD2A and LGMD2B), and the two markers were found unaffected.

**Table 1:** Features of the analyzed cell lines.

CELLLINES	AGE ATBIOPSY/GENDER	SITE OFBIOPSY	MOLECULARDIAGNOSIS	SIZE OFD4Z4ARRAY	HAPLOTYPE*	PD^a^	MICROARRAYASSAY**	qRT-PCR**
FSHD-1S1	35 / M	Quadricepsfemoris	FSHD-1	23 kb	4qA161–4qB168	7		X
FSHD-1S2	5 / M	Quadricepsfemoris	FSHD-1	6–9 kb	4qA161–4qB164	4	X	X
FSHD-1S3	71 / F	Quadricepsfemoris	FSHD-1	27,5 kb	4qA161–4A163	5	X	X
FSHD-1S4	55 / F	Quadricepsfemoris	FSHD-1	29 kb	4qA161–4qA161	3	X	X
FSHD-1S5	75 / F	Quadricepsfemoris	FSHD-1	25 kb	N.D.	4		X
FSHD-1S6	33 / M	Quadricepsfemoris	FSHD-1	27,5 kb	N.D.	5		X
FSHD-1S7	17 / F	Quadricepsfemoris	FSHD-1	17 kb	N.D.	4		X
FSHD-2S1	12 / M	Quadricepsfemoris	FSHD-2	>38 kb	4qA161–4qB168	4	X	X
FSHD-2S2	17 / M	Quadricepsfemoris	FSHD-2	>38 kb	4qA161–4qA161	3	X	X
CN-1	62 / F	Quadricepsfemoris	control	-	4qA161–4qB164	5	X	
CN-2	77 / M	Quadricepsfemoris	control	-	4qA161–4qB164	4	X	
CN-3	55 / F	Quadricepsfemoris	control	-	4qA168–4qB164	4	X	X
CN-4	60 / M	Quadricepsfemoris	control	-	4qA161–4qB163	3		X
CN-5	20 / M	Quadricepsfemoris	control	-	N.D.	2		X
CN-6	28 / F	Quadricepsfemoris	control	-	4qA161–4qB163	5		X
CN-7	37 / F	Quadricepsfemoris	control	-	4qA161–4qB164	6		X
CN-8	49 / F	Quadricepsfemoris	control	-	N.D.	10		X

N.D. Not determined.

a Population doubling (PD).

*Sequence-lengh polymorphism (SSLP) located 3,5 kb proximal to D4Z4 [7].

**Cell lines used in microarray assay and/or qRT-PCR.

### Total RNA extraction and qRT-PCR analysis

Total RNA was isolated from sub confluent cell cultures using the RNeasy Mini Kit (Qiagen), and the purified RNA was treated with RNase-free DNase (Qiagen) to remove any residual DNA. Purified RNA was quantified by NanoDrop spectrophotometry (Thermo Scientific). Quantitative RT-PCR (qRT-PCR) analysis was performed on an IQ™5 Multicolor Real-Time PCR Detection System (Biorad) by TaqMan® Gene Expression Assays (Applied Biosystem) (Table 2), or SYBR Green (Biorad) (Table 3). Each amplicon was analyzed in duplicate in 96-well optical plates. For TaqMan® Gene Expression Assays, typical 20 µl reactions contained 10 µl IQ multiplex supermix (Biorad), 1 µl 20x TaqMan Gene Expression Assay Mix (containing unlabeled PCR primers and FAM dye-labeled MGB probe; Applied Biosystem), and 20 ng of cDNA. SYBR Green qRT-PCR was performed as previously described [17]. For the TaqMan® Assay Thermal cycling conditions were 2 min at 95°C, followed by 40 cycles at 95°C for 10 s and 60°C for 30 s. PCR conditions for SYBR Green assay are described in Table 3. Each experiment was performed on three independent RNA extractions of the same sample. For SYBR Green assays, standard curves for each amplicon were generated using cDNA derived from a serial 5 fold dilution of human muscle cDNA, derived from a commercial RNA (Ambion). Glyceraldehyde-3-phosphate dehydrogenase (GAPDH), hypoxanthine phosphoribosyl transferase-1 (HPRT1) and polymerase RNA II DNA-directed polypeptide A (POLR2A) were initially tested as house-keeping genes. Since they displayed a similar expression range (data not shown), we decided to use only GAPDH. Thus results were normalized to PCR product for GAPDH using the comparative 2^-ΔΔCt^ method and are presented as fold change (FC) [19].

**Table 2:** TaqMan RT–PCR primers and probes.

Transcript	Primer (5′–3′)	Probes (Reporter – Quencher)
GAPDH	Fw - cccttcattgacctcaactacatg	TEXAS RED - BHQ-2 (Sigma)
	Rw - tgggatttccattgatgacaagc	
POLRA2	Fw- gcaccacgtccaatgacat	HEX - BHQ-1 (Sigma)
	Rw- gtgcggctgcttccataa	
HPRT1	Fw- agactttgctttccttggtcagg	JOE - TAMRA (Sigma)
	Rw- gtctggcttatatccaacacttcg	
KIF18A	Hs00229692_m1	FAM - BHQ-2 (Applied Biosystems)
CDC6	Hs00154374_m1	FAM - BHQ-2 (Applied Biosystems)
E2F7	Hs00403170_m1	FAM - BHQ-2 (Applied Biosystems)
SUV39H1	Hs00162471_m1	FAM - BHQ-2 (Applied Biosystems)
DCLRE1B	Hs00224566_m1	FAM - BHQ-2 (Applied Biosystems)
MSH2	Hs00953523_m1	FAM - BHQ-2 (Applied Biosystems)
SDR	Hs00190538_m1	FAM - BHQ-2 (Applied Biosystems)
LAMA4	Hs00935293_m1	FAM - BHQ-2 (Applied Biosystems)
SOD2	Hs00167309_m1	FAM - BHQ-2 (Applied Biosystems)
PTPRN	Hs00160947_m1	FAM - BHQ-2 (Applied Biosystems)

**Table 3:** SYBR Green Assay primer pairs and PCR efficiency.

Transcript	Primer (5′–3′)	Correlation coefficient (R∧2)
		Efficiency of reaction (E)
PAX3	Fw- ggagactggctccatacgtc	E = 99,0%
	Rw- caaattactcaaggacgcgg	R∧2 = 0,924
MYOD1	Fw- cggcggaactgctacgaag	E = 99,5%
	Rw- gcgactcagaaggcacgtc	R∧2 = 0,990
MYOG	Fw- tcaaccaggaggagcgtgac	E = 97,0%
	Rw- tgtagggtcagccgtgagca	R∧2 = 0,979
MYH2	Fw- ggaccaactgagtgaactgaaa	E = 95,8%
	Rw- ttgcctcttgataactgagacac	R∧2 = 0,908

The statistical analysis was performed using a two-tailed Student's *t*-test and the error bar is ERR.STD.

### Microarray Assay

RNA quality and quantity were assessed using Agilent 2100 Bioanalyzer (Agilent Technologies) and NanoDrop ND-1000 Spectrophotometer (Thermo Fisher Scientific), respectively. 1 µg of total RNA was subjected to ribosomal RNA removal using RiboMinus human/mouse transcriptome isolation kit (Invitrogen), then cDNA was synthesized using Whole-Transcript Sense Target Labeling Assay (Affymetrix®), following manufacturer's procedure. Fragmented biotin-labeled cDNAs were hybridized to Affymetrix® human exon 1.0 ST arrays at 45°C for 17 hours, as described in Affymetrix® Users Manual. Washing and staining steps were carried out using GeneChip Fluidics Station 450, then the arrays were scanned in the Affymetrix® GeneChip® scanner 3000 7G. Affymetrix® GeneChip® operating software was used for acquisition, management and initial processing of the expression data, while arrays quality control was performed using Affymetrix® Expression Console™.

### Microarray data analysis

Expression analysis of microarray experiments was performed with Raw Affymetrix data (".CEL" files) were background adjusted, preprocessed and normalized using RMA procedure. The analyses were performed using R statistical environment (www.r-project.org) with Bioconductor libraries for microarray data analysis (www.bioconductor.org). Custom probeset definitions were adopted [20]: library version 11. In particular, the ENSEMBL based probeset definition was used to obtain gene expression data. Expression data matrices were filtered to select only custom probeset including at least 4 probes. Differential expression was evaluated using limma package (www.bioconductor.org) considering the contrasts of interest between selected groups of samples. Moderated statistics were computed using limma empirical Bayes adjustment for standard errors. Gene probesets with P<0.01 and FC>2 were selected in FSHD-1 assay, whereas P<0.001 and FC>2 were used in FSHD-2, in the attempt to overcame problems due to the small sample size analyzed. All data discussed in this publication are MIAME compliant; all data have been deposited in Gene Expression Omnibus (NCBI) and are accessible through GEO Series accession number GSE26061.

Functional classification analysis of the differentially expressed probes was performed with DAVID Bioinformatics Resource 6.7 (National Institute of Allergy and Infectious Disease (NIAD), NIH (http://david.abcc.ncifcrf.gov) [21]–[22], and by Gorilla [23]. We considered GeneOntology functional classes that had a Fisher exact *p*-value (EASE score) <0.05.

### miRNA target prediction

miRNA target prediction was obtained with microRNA.org (http://www.microrna.org/microrna/home), PicTar (http://pictar.mdc-berlin.de/), miRNAMap (http://mirnamap.mbc.nctu.edu.tw), TargetScanHuman 5.1 (http://www.targetscan.org), Microcosm Target (http://www.ebi.ac.uk/enright-srv/microcosm/htdocs/targets/v5).

## Results

### Expression profiles of FSHD-1 and FSHD-2 myoblasts and myotubes

**Figure 1:**
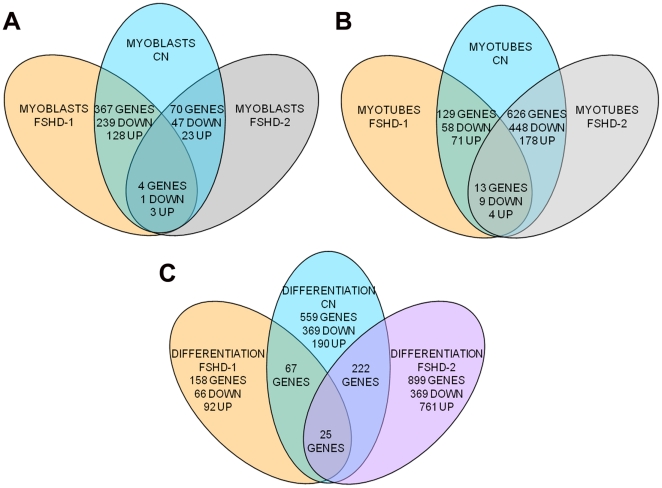
Venn diagrams showing overlapping and non-overlapping counts of genes differentially expressed. A): FSHD-1 and FSHD-2 myoblasts, in respect to controls; 367 genes were up- (128) or down- (239) regulated in FSHD-1 myoblasts and 70 genes were up- (23) or down- (47) regulated in FSHD-2 myoblasts. Four (3 up and 1 down) genes were deregulated in both cell lines. B): FSHD-1 and FSHD-2 myotubes, in respect to controls; 129 genes were up- (71) or down- (58) regulated in FSHD-1 myotubes and 626 genes were up- (178) or down- (448) regulated in FSHD-2 myotubes. Thirteen (4 up and 9 down) genes were deregulated in both cell lines. C): FSHD-1 and FSHD-2 differentiation processes, in respect to the differentiation of control cells; 559 genes were modulated in the differentiation of CN cells, 158 in the differentiation of FSHD-1 and 899 of FSHD-2, cells. FSHD-1 and CN differentiation processes shared 67 entries, whereas FSHD-2 and CN differentiation processes shared 222 entries. FSHD-1 and FSHD-2 differentiation processes shared 25 deregulated genes. FC>2 and p-value <0.01 and <0.001 for FSHD-1 and FSHD-2, respectively.

We analyzed by microarray the expression profile of human primary myoblasts obtained from three FSHD-1 and two FSHD-2 patients, and three healthy controls (CN). To evaluate the molecular perturbation of FSHD upon muscle differentiation, we compared patients and CN proliferating myoblasts as well as the corresponding myotubes obtained after 8 days of cell differentiation. Moreover we analyzed gene expression variations in the differentiation processes of FSHD samples and compared them to that observed in control cells (Fig. 1). Setting the criteria described in Material and Methods (FC>2 and p-value <0.01 and <0.001 for FSHD-1 and FSHD-2, respectively), FSHD-1 and FSHD-2 proliferating cells showed a total of 367 (239 down and 128 up) and 70 (47 down and 23 up) deregulated probes as compared to controls respectively, sharing only 4 genes (1 down and 3 up) (Fig.1A). The same analysis performed on the corresponding myotubes (Fig.1B) evidenced a total of 129 (58 down and 71 up) in FSHD-1 and 626 (448 down and 178 up) in FSHD-2 deregulated probes, respectively. Also in this case the number of shared genes was very low (13 genes, 9 down and 4 up). The four gene lists are reported in Table S1A-D. Analyzing the gene expression during the differentiation processes we obtained a total of 559 genes modulated in CN cells, 158 in FSHD-1 and 899 in FSHD-2 cells. The FSHD-1 differentiation process shared with the CN one only 67 entries, whereas FSHD-2 and CN differentiation processes shared 222 entries (Fig.1C). Furthermore, the two pathological differentiation processes shared 25 deregulated genes.

Regarding the 4q35 chromosome region, the analysis did not reveal a significantly altered pattern of gene expression, in both FSHD-1 and FSHD-2 samples; exceptions were represented by three genes (SNX25, ANKRD37 and SORBS2, located approximately 4 Mb upstream to the D4Z4 array) found down-regulated only in FSHD-1 myotubes (Table S1C).

**Figure 2:**
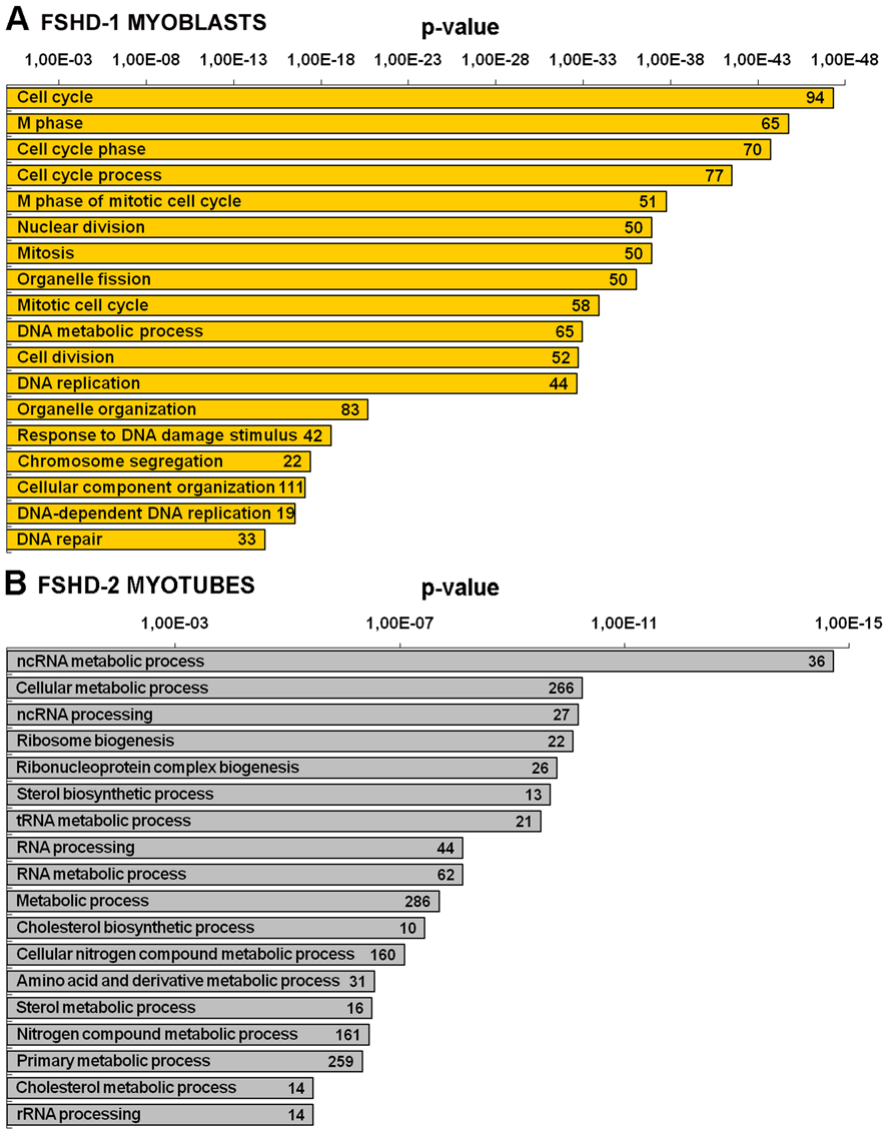
Selected gene classifications according to biological processes. Biological processes significantly enriched in the set of genes identified by microarray as up- or down-regulated in A) FSHD-1 myoblasts, and B) in FSHD-2 myotubes, and categorized in the DAVID program. Numbers in the bars indicate the number of genes assigned to each gene ontology term. p-value <0.05.

The probes identified by microarray as up- or down-regulated in FHSD-1 and FSHD-2 cells were categorized in the DAVID program (see criteria in Materials and Methods). As shown in Fig. 2A, the most severely affected biological processes in FSHD-1 myoblasts in respect to control cells were mainly linked to cell cycle (94 genes, 35% of total deregulated probes), particularly M-phase (65 genes out of 94), and to DNA metabolic process (65 genes) and replication (44 genes). More precisely, these classes that represent the most significant ones (p-value <10^−30^) are essentially composed by down-regulated genes. In these biological categories we found, all down-regulated, seven cell division cycle genes (CDC, involved in G1/S and G2/M transitions), eight minichromosome maintenance complex components (MCM, required for the entry in S phase and cell division), two cyclins (CCNA2 and CCNF), two cyclin-dependent kinases (CDK1 and CDK2), several factors involved in DNA replication (four DNA-dependent DNA polymerases, one primase and one helicase) and repair (MSH2, DCLRE1B, BRCA1 and BRCA2), eight kinesins (KIF, involved in spindle formation and the movements of chromosomes during mitosis), and five centromere proteins (CEMP). Among the up-regulated genes, of particular interest was GAS1, involved in growth arrest. Furthermore, the careful inspection of the deregulated gene list of FSHD-1 myoblasts allowed the identification of several entries previously reported to be involved in FSHD-1 and in the myogenic program. We found the down-regulation of two genes (SUV39H1 and HMGB2) involved in chromatin conformation mechanism, and the up-regulation of two myogenic markers (PAX3 and MYOD1) and of SOD2 involved in oxidative stress response (Table S1A). All together the above results suggest the occurrence in FSHD-1 myoblasts of a damage in cell cycle progression, and in myogenic differentiation.

Conversely, the categories of biological processes identified as more severely affected in FSHD-2 myoblasts (Table S1B) did not show very significant p-values (>10^−3^) and were mainly associated to extracellular structure organization and system development. Also the functional analysis of FSHD-1 myotubes (Table S1C) identified a limited number of biological processes, without very significant p-values (approximately 10^−4^), essentially involved in transport and biosynthetic processes.

Differently from what observed in FSHD-1 myotubes, FSHD-2 differentiated cells showed that the most significantly affected biological process (p-value up to 10^−14^) was related to ncRNA metabolic process, including ribosome biogenesis (22 genes) and tRNA metabolic process (21 genes). Others significantly affected categories regarded sterol (13 genes) and amino acid metabolic processes (31 genes) (Fig. 2B). Considering the tRNA metabolic process, about 50% of all entries were tRNA synthases (ARSs). Interestingly, in respect to cellular components the DAVID program highlighted in FSHD-2 myotubes as the main deregulated the nucleolus (55 genes) and the mitochondrion (53 genes); almost all the deregulated genes in these categories were down-regulated. The mitochondrion showed the deregulation of many ribosomal proteins (seven MRPs), four genes involved in respiratory chain, including ATP synthase, six transporters (i.e. SLC, TIMM and TOMM), and three genes involved in fission and fusion (DNM1L, MTFR1, and MFN2). Two entries (GSR and GPX4) involved in the response to oxidative stress were also found down-regulated. In addition the inspection of the gene list containing all the deregulated genes (Table S1D) showed the down-regulation of five eukaryotic translation initiation factors (EIFs). Thus, FSHD-2 myotubes were principally affected in functions related to protein synthesis, to sterol biosynthetic process and to energetic metabolism. The complete lists of all the significant deregulated biological categories of the four analyzed cell typologies (FSHD-1 and FSHD-2 myoblasts and myotubes) are reported in Table S2A-D.

**Figure 3:**
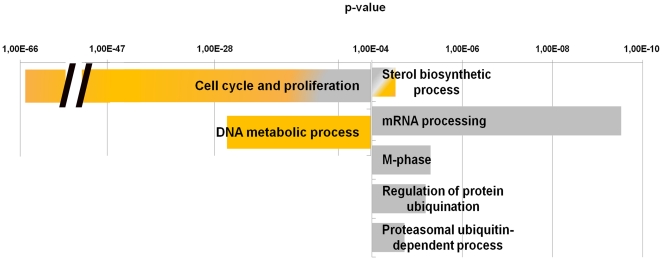
Selected gene classifications according to biological processes of genes regulated in the three differentiation programs. Diagram showing the biological processes significantly enriched in the set of genes differentially expressed in the differentiation processes of FSHD-1 (yellow bars) and FSHD-2 (grey bars) cells in respect to control. Bar on the left indicated the biological process modulated in control but not in FSHD-1 (yellow bar), and FSHD-2 (grey bars) cells, whereas bars on the right indicated the biological processes modulated in FSHD-1 (yellow bar) and FSHD-2 (grey bars) but not in control cells. All bars group many related GO categories. p-value <10^−4^.

Another approach to investigate the gene deregulation in FSHD cells is to analyze the gene chip results in the context of the differentiation process. This could be obtained by categorizing in the DAVID program the variation in gene expression profile obtained analyzing the FSHD-1 and FSHD-2 differentiation processes subtracted with the variation showed by the control cells differentiation. The result of these analyses is schematized in Fig.3, where on the left is reported the biological process not modulated in FSHD-1 (yellow bar) and in FSHD-2 (grey bar) cells, in respect to control, whereas on the right the biological processes modulated in FSHD-1 (yellow bar) and in FSHD-2 (grey bars), but not in control cells. Both pathological differentiation processes showed as mainly deregulated categories those already derived in FSHD-1 myoblasts (cell cycle and proliferation) and FSHD-2 myotubes (RNA processing) (Figs. 2 and 3). In addition, this analysis evidenced a slight damage of cell cycle also in FSHD-2 and of the proteasomal ubiquitin-dependent processes. Interestingly, FSHD-1 and FSHD-2 cells showed the common deregulation of five genes involved in cholesterol metabolic process; four genes (HMGCR, DHCR7, DHCR24 and IDI1) implied in cholesterol biosynthesis, were up-regulated in FSHD-1 and down-regulated in FSHD-2, and one gene (ABCA1) involved in the efflux of cholesterol from the cell, was down-regulated in FSHD-1 and upregulated in FSHD-2. The complete lists of all the significant deregulated biological categories of the three differentiation processes are reported in Table S3.

### MicroRNA deregulation

The gene expression profile analysis of FSHD-1 and FSHD-2 myoblasts and myotubes also evidenced a total of six deregulated miRNAs. Two miRNAs, mir-23b and mir-133a-1, were upregulated in FSHD-1 and FSHD-2 myoblasts, respectively; FSHD-1 myotubes showed the down-regulation of one miRNA (mir-186), whereas in FSHD-2 myotubes four miRNAs (mir-149, mir-15a, mir26a-2, and mir23b) were up-regulated. Furthermore, mir-23b was found deregulated both in FSHD-1 myoblasts and FSHD-2 myotubes. The derived list of deregulated miRNAs was then analyzed for the predicted targeted genes (see Materials and Methods). To this aim we used five different softwares, and only the gene targets predicted by at least three softwares and showing an opposite trend of deregulation in the gene chip analysis were considered. By using these criteria, the miRNAs 133a-1 was not further considered. Table 4 reports the five miRNAs and the corresponding predicted targeted genes found deregulated in FSHD-1 myoblasts and myotubes and in FSHD-2 myotubes, with the corresponding fold change and p-value. Interestingly, some of the predicted targets of the miRNAs deregulated in FSHD-2 myotubes could be included into the functional categories represented in the Gene Ontology analysis of these samples, such as RNA biosynthesis (NFIB and ZNF410) and cholesterol biosynthesis (SC4MOL). One gene (PRKAR2A) was targeted by more then one miRNA (asterisked in Table 4).

**Table 4:** miRNAs significantly dysregulated in FSHD-1 and FSHD-2 myoblasts and myotubes, with the corresponding predicted gene targets.

	miRNA		GENE TARGET		
CELLLINES	GENESYMBOL	FC (P-VALUE)	GENESYMBOL	FC (P-VALUE)	FUNCTION
FSHD-1myoblasts	hsa-mir-23b	3,4 (6,90E-03)	HMGB2	−2,1 (1,91E-03)	Chromatin conformation
FSHD-1myotube	hsa-mir-186	−2,3 (2,09E-03)	HAS2	3,8 (1,48E-03)	Biosynthesis of extracellular matrix
FSHD-2myotubes	hsa-mir-149	3,18 (4,22E-05)	NFIBPRKAR2A*	−5,77(2,41E-05)−2,48 (2,43E-04)	RNA biosynthesisSignal transduction
FSHD-2myotubes	hsa-mir-15a	6,19 (6,07E-05)	IARSCOPS7APRKAR2A*ARL2	−3,03 (8,95E-06)−2,23 (1,35E-05)−2,48 (2,43E-04)−2,54 (4,76E-04)	Protein synthesisSignal transductionSignal transductionTransport
FSHD-2myotubes	hsa-mir-26a-2	2,64 (5,80E-04)	EPC2ZNF410FAM55CSC4MOL	−2,95 (5,67E-05)−2,33 (1,91E-04)−3,30 (3,97E-04)−7,49 (1,13E-05)	Chromatin conformation; regulation of transcriptionRNA biosynthesisUnknownCholesterol byosinthesis
FSHD-2myotubes	hsa-mir-23b	6,57 (6,79E-04)	EPS15ENTPD5	−2,28 (9,07E-04)−2,27 (1,67E-04)	TransportNucleotide metabolism

*The asterisk indicates the only gene targeted by more than one miRNA.

### Validation of microarray results by Quantitative Real–Time PCR

**Figure 4:**
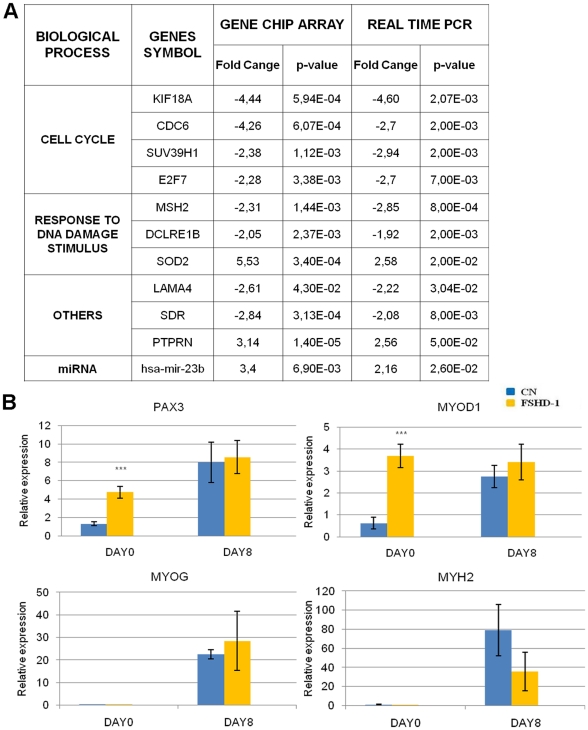
Real-Time PCR validation of FSHD-1 microarray data. A: Table reports the genes analyzed in qRT-PCR with fold-change and p-value. The data obtained in the FSHD-1 myoblasts gene chip array and the biological processes identified by the DAVID program, are also reported. The analysis was performed on seven FSHD-1 and six CN myoblast cell lines. B) Bar diagrams show relative expression of PAX3, MYOD1, MYOG and MYH2 in control and FSHD-1 myoblasts (day 0) and myotubes (day 8) relative to GAPDH. *** p-value < ,001.

To confirm the FSHD-1 microarray data we focused our attention on some genes contained in the most enriched categories evidenced by GO analysis. The chosen representative genes were validated by multiplex Real–Time assay performed with Taqman® probes on seven FSHD-1 in comparison to six healthy controls (for a description of the used cell lines see Table 1 in Materials and Methods). The analyzed genes comprised: E2F7 (negative regulator of cell cycle progression), CDC6 (DNA replication), KIF18A (chromosome segregation) and SUV39H1 (histone methylation), MSH2, DCLRE1B, SOD2 (DNA damage and repair), LAMA4 (extracellular matrix), SDR (membrane raft) and PTPRN (cell growth and differentiation). mir 23-b was also analyzed. As shown in Fig.4A, the results of the Real–Time assay confirmed the data of the array. In fact all genes tested were regulated in the same direction with both methods. The data relative to the FSHD-2 genechip analysis were not validated by qRT-PCR due to the unavailability of other FSHD-2 cell lines in addition to those used for microarray experiments.

Furthermore, since the analysis of the FSHD-1 myoblasts gene chip array evidenced a slight up-regulation of the transcription factor PAX3 (P 1×10^−2^; FC 2.17), a molecule involved in developmental myogenesis, and of MYOD (P 1,01×10^−2^; FC 2.39, Table S1A), we also evaluated by real-time PCR the gene expression level of these and of two other myogenic markers (MYOG and MYH2) not significantly deregulated in the chip assay, using cDNAs from FSHD-1 cells before and after differentiation (day 0 and 8).

As shown in Fig.4B, the expression level of the four myogenic markers (PAX3, MYOD1, MYOG and MYH2) in control and FSHD-1 cells showed a general trend of up-regulation upon differentiation. In FSHD-1 proliferating cells compared to control, PAX3 and MYOD1 were, respectively almost four and six-fold up-regulated, respectively; conversely, MYOG and MYH2 mRNA levels were undetectable, both in control and FSHD-1 cells. Upon differentiation, all markers showed comparable levels of mRNA expression in FSHD-1 and control cells. These results confirm the general trend of differentiation exhibited by both control and FSHD-1 cells and that significant differences are only present in FSHD-1 proliferating cells for PAX3 and MYOD mRNAs levels. Regarding FSHD-2 cells the gene chip assay data evidenced for these four myogenic markers an expression level similar to that of control cells both before and after cell differentiation (Table S1B and S1D).

## Discussion

In this paper we have compared the expression profiles of FSHD-1 and FSHD-2 precursor cells in regard to healthy controls before and after myogenic differentiation. In our knowledge, this is the first report that uses human 4q-linked and non 4q-linked (or phenotypic) FSHD primary myoblasts and their in vitro differentiation to investigate global gene deregulation characterizing cells deriving from FSHD patients with a different genetic defect, but with a very similar phenotypic manifestation of the disease [24]. Although the in vitro differentiation of myoblasts does not involve many of the complex series of events known to be important in vivo, such as activation of quiescent satellite cells (stem cell), maturation of the myotubes into muscle fibers and the innervations of the fibers, the cell system we used could be useful in the attempt to derive global gene expression deregulation characterizing the early stages of myogenic differentiation without interferences represented by cell contamination, inflammation or muscle regeneration as found in studies of biopsies.

It is noteworthy that only two FSHD-2 cell lines were available for the chip analysis; although this represents a small sample size we decided anyhow to include them in our analysis since this type of FSHD cell has never been analyzed; thus to render the data more significant we decided to use a lower p value (>0.001) than that used for FSHD-1 (>0.01).

A gradient of altered gene expression throughout the 4q35 chromosome linked to D4Z4 contraction has been proposed as a model for the molecular pathogenesis of FSHD-1 [15]. Our results did not evidence such a correlation in both FSHD-1 and FSHD-2 cells. Only three genes (SNX25, ANKRD37 and SORBS2) located approximately from 4 to 5 Mb proximal to the D4Z4 array showed in FSHD-1 myotubes a significant down-regulation. Interestingly, one of these genes (ANKRD37) was also found deregulated in muscle biopsies from FSHD-1 patients [25]. Absence of significant gene expression alteration throughout the 4q35 region agrees with the data previously reported by Winokour et al. (2003) [26] and Osborne et al. (2007) [27] on muscle biopsies, thus excluding a position effect model for FSHD. However, we can not exclude the possibility that some of the 4q35 genes (i.e. FRG1) might be transiently deregulated during intermediate steps of the differentiation process [17].

However, significant results concerning the altered biological processes of the pathological cells were obtained, by deriving in regard to controls the global deregulation of gene expression in FSHD-1 and FSHD-2 myoblasts and myotubes and by comparing the two pathological differentiation processes to the normal one. By combining the two approaches, we derived that gene deregulation was essentially a feature of FSHD-1 proliferating cells and of FSHD-2 differentiated cells. FSHD-1 myoblasts showed a highly significant gene deregulation linked to cell cycle control essentially affecting G1/S and G2/M transitions. These results are in agreement with previous data derived by the analysis of FSHD-1 cells, and showing the up-regulation of p21, known to arrest progression at G1/S interface, and of WEE1, a negative regulator of entry into mitosis (G2/M transition) [16], [26].

Furthermore, FSHD-1 myoblasts showed the up-regulation of PAX3, a key upstream regulator of the myogenic program: PAX3 up-regulates the myogenic determination gene MYOD1 that, in turn regulates MYOG expression [28]. However, while in embryonic tissues the ability of PAX3 to activate the myogenic program is well documented [29]-[30], in adult-derived cells this effect is still under discussion.[28], [31]–[32].

In our system, the found premature up-regulation of MYOD1 mRNA could be ascribed to PAX3 mRNA up-regulation; furthermore, as previously reported [33], also in our cellular system MYOD-mediated induction of myogenesis is accompanied by the down-regulation of cyclins. Thus, PAX3 up-regulation might contribute to the early cell cycle arrest shown by FSHD-1 myoblasts. In spite of the up-regulation of MYOD1 mRNA, FSHD-1 proliferating cells did not show the occurrence of later marker of myogenic differentiation, such as myogenin and sarcomeric myosin. This could probably due to the absence of other required transcription factors such as myogenic enhancer factors (MEFs), essential for muscle differentiation. Thus, FSHD-1 cells seem to be characterized by a premature and partial activation of the myogenic program that could be related to the observed defect in cell cycle progression.

Remarkably, other two genes SUV39H1 and HMGB2 both involved in chromatin remodeling were down-regulated in FSHD-1 myoblasts. SUV39H1 is a histone methyl-transferase involved in D4Z4 H3K9me3 [34], whereas HMGB2 is part of a multi-protein complex shown to bind a 27bp binding element (DBE) within D4Z4 units [15], [35]. In normal cells both gene activities, in association with other factors, may play an important role in the establishment and maintenance of the higher order chromatin structure of the D4Z4 array (facultative heterochromatin). In FSHD-1 cells, the down-regulation of SUV39H1 and HMGB2 genes could correlate with the hypothesized more open chromatin conformation of the contracted 4q alleles [8], [10].

Thus, in addition to the early partial activation of the myogenic program, proteins involved in chromatin organization are also modulated in FSHD-1 samples, suggesting that their absence might contribute to the epigenetic defect of the D4Z4 array.

Conversely, FSHD-2 cells were characterized by a significant alteration of gene expression only after the *in vitro* transition from myoblasts to myotubes. Effectively, FSHD-2 myotubes showed the deregulation of genes essentially involved in non-coding RNA metabolism and in nucleolus organization, implied in protein synthesis. FSHD-2 myotubes also showed mitochondrial abnormalities, including energy production, response to oxidative stress and mitochondrial dynamics. Mitochondrial abnormalities and dysfunction in protein synthesis have been also reported for other muscular dystrophies [36]-[39].

FSHD-2 differentiation analysis also evidenced the deregulation of the cell cycle and of proteasomal ubiquitin-dependent process. Importantly, ubiquitin-dependent proteolysis has been suggested to govern terminal muscle differentiation by coordinating cellular division and differentiation [40].

Interestingly, both FSHD-1 and FSHD-2 cells were affected in sterol biosynthetic process, showing the deregulation, although in the opposite direction, of the same genes. The alteration of cholesterol homeostasis could primarily cause cell damage in membranes lipid rafts, where different proteins are incorporate (e.g. GPI-anchored and cholesterol-linked proteins), and in caveolae a subclass of rafts [41]. It was previously reported that caveolae structure alteration could affect myotube formation [42]–[43], and that FSHD-1 biopsies are characterized by the impairment of biological processes involved in the synthesis of GPI anchored proteins [25].

In normal cells reactive oxygen species (ROS) generation is counterbalanced by the action of antioxidant enzymes, such as mitochondrial superoxide dismutase (SOD2) and of those involved in glutathione metabolism. The found deregulation of SOD2 in FSHD-1 myoblasts and of glutathione reductase (GSR) and peroxidase (GPX4) in FSHD-2 myotubes could suggest for both FSHD manifestations the occurrence of a similar increased susceptibility to oxidative stress. The deregulation of enzymes involved in oxidative stress resistance and the consequent increased susceptibility to oxidative stress have been already reported for FSHD-1 myoblasts and biopsies [16], [26]–[27], [44].

Finally, both FSHD-1 and FSHD-2 cells showed the involvement in the gene deregulation network of some microRNAs (miRNA), a class of molecules previously shown to play an important role in the regulation of muscle development [45]. Among a total of five miRNAs found deregulated in the present work, two (mir186 and mir15a) were previously reported to be commonly deregulated in more than three (including FSHD) types of muscular disorders [46]. The remaining three miRNAs were detected in FSHD-1 myoblasts (mir-23b) and in FSHD-2 myotubes (mir-149, mir-26a2 and mir-23b). Interestingly, one predicted target of the supposedly FSHD-specific miRNA 23b is a gene involved in the chromatin conformation of the 4q D4Z4 array (HMGB2 down-regulated in FSHD-1 myoblasts) [15].

Although future work is certainly needed to confirm the herein derived observations, taken together our results seem to recapitulate previously reported defects of FSHD-1, and to add new insights into the gene deregulation characterizing both FSHD-1 and FSHD-2. In general, FSHD-1 cells showed an alteration of cell cycle control, a defect in cholesterol homeostasis and presumably in the mitochondrial capacity to buffer oxidative stress. With the exception of cholesterol homeostasis, FSHD-2 cells shared all these features by deregulating different genes. FSHD-2 cells also showed a general deregulation of protein synthesis and degradation. In this regard, proteasome ubiquitin-dependent protein degradation could be viewed as an impairment in exit from the cell cycle. Thus both FSHD manifestations presented cellular deficiencies that do not arise from a 4q position effect mechanism, but rather from a general alteration of gene expression in which miRNA deregulation may play a role.

## Supporting Information

Table S1 List of differentially expressed probes in FSHD-1 and FSHD-2 myoblasts (A and B), and in FSHD-1 and FSHD-2 myotubes (C and D).
(XLS)

Table S2 DAVID classification of biological processes significantly enriched in the set of genes differentially expressed in FSHD-1 and FSHD-2 myoblasts (A and B) and in FSHD-1 and FSHD-2 myotubes (C and D).
(XLS)

Table S3 DAVID classification of biological processes significantly enriched in the set of genes differentially expressed during the differentiation process of CN (A), FSHD-1 (B) and FSHD-2 (C) cells.
(XLS)
